# Characterization of Two VAO-Type Flavoprotein Oxidases from *Myceliophthora thermophila*

**DOI:** 10.3390/molecules23010111

**Published:** 2018-01-05

**Authors:** Alessandro R. Ferrari, Henriëtte J. Rozeboom, Aniek S. C. Vugts, Martijn J. Koetsier, Robert Floor, Marco W. Fraaije

**Affiliations:** 1 Molecular Enzymology, Groningen Biomolecular Sciences and Biotechnology Institute, University of Groningen, 9747AG Groningen, The Netherlands; a.ferrari88@gmail.com (A.R.F.); h.j.rozeboom@rug.nl (H.J.R.); 2 Dupont Industrial Biosciences, 6709PA Wageningen, The Netherlands; aniek.vugts@dupont.com (A.S.C.V.); martijn.koetsier@dupont.com (M.J.K.); robert.floor@dupont.com (R.F.)

**Keywords:** oxidase, bicovalent, crystal structure, flavin adenine dinucleotide (FAD)

## Abstract

The VAO flavoprotein family consists mostly of oxidoreductases harboring a covalently linked flavin cofactor. The linkage can be either monocovalent at position 8 with a histidine or tyrosine or bicovalent at position 8 with a histidine and at position 6 with a cysteine. Bicovalently bound flavoproteins show a preference for bulkier substrates such as oligosaccharides or secondary metabolites. The genome of the thermophilic fungus *Myceliophthora thermophila* C1 was found to be rich in genes encoding putative covalent VAO-type flavoproteins. Enzymes from this fungus have the advantage of being rather thermostable and homologous overexpression in *M. thermophila* C1 is feasible. Recently we discovered a new and VAO-type carbohydrate oxidase from this fungus: xylooligosaccharide oxidase. In this study, two other putative VAO-type oxidases, protein sequence XP_003663615 (MtVAO615) and XP_003665713 (MtVAO713), were expressed in *M. thermophila* C1, purified and characterized. Enzyme MtVAO615 was found to contain a bicovalently bound FAD, while enzyme MtVAO713 contained a monocovalent histidyl-bound FAD. The crystal structures of both proteins were obtained which revealed atypical active site architectures. It could be experimentally verified that both proteins, when reduced, rapidly react with molecular oxygen, a hallmark of flavoprotein oxidases. A large panel of alcohols, including carbohydrates, steroids and secondary alcohols were tested as potential substrates. For enzyme MtVAO713 low oxidase activity was discovered towards ricinoleic acid.

## 1. Introduction

Oxidases are enzymes capable of performing selective oxidative reactions using molecular oxygen as electron acceptor. They represent valuable and cost-effective biotechnological tools for industrial applications since they do not require expensive coenzymes but just molecular oxygen. To catalyze oxidations, oxidases typically employ a copper ion or a flavin as cofactor. Known examples of the former are galactose oxidase and laccases. The flavin-containing group of oxidases is more abundant. Oxidases in this category contain mostly a flavin adenine dinucleotide (FAD) as prosthetic group with a minority containing flavin mononucleotide (FMN). Based on sequence homology and the available structural information, six families of flavoprotein oxidases have been identified [1].

The FAD/FMN cofactor can be bound to the protein either non-covalently or covalently. The elucidation of the crystal structure of vanillyl alcohol oxidase (VAO) showed for the first time the FAD cofactor covalently bound to a protein via a histidine residue [2]. Members of the VAO flavoprotein family share a common overall structure which is composed of two domains: a conserved FAD binding domain that binds the adenine part of the FAD cofactor and a variable cap domain that covers the isoalloxazine ring of the cofactor and forms the substrate binding pocket [3]. In 2005 the crystal structure of another member of the VAO-type family, glucooligosaccharide oxidase (GOOX), was elucidated. This revealed for the first time a flavin cofactor covalently linked to two amino acids [4]. By having the FAD cofactor anchored by two covalent bonds, these bicovalent flavoproteins proteins seem to have evolved a rather open active site which allows them to accept bulky molecules as substrates [5]. It has also been shown that covalent tethering of the FAD to the protein increases the flavin redox potential, with bicovalent flavoproteins having the highest redox potentials [5]. With such high redox potentials, molecular oxygen is one of the few natural electron acceptors that covalent flavoproteins can use. This is probably the reason why most covalent VAO-type flavoproteins are oxidases [1].

Among the VAO-type oxidases, a great variety of substrates are accepted [6]. Some oxidases are part of gene clusters involved in tailoring secondary metabolites. Therefore they accept relatively complex biomolecules as substrates. Examples of these are Dbv29 that acts on the precursor of the glycopeptide A40926 [7], aclacinomycin oxidase which catalyzes the oxidation of a C-O bond and the dehydrogenation of the sugar moiety of aclacinomycin precursors [8], and the tirandamycin oxidase that catalyzes the oxidation of a secondary alcohol moiety of a tirandamycin intermediate [9]. Also the recently discovered subfamily of VAO-type carbohydrate oxidases, such as xylooligosaccharide oxidase (XylO) [10], chitooligosaccharide oxidase (ChitO) [11] or GOOX [12], act preferably on bulky oligosaccharides while monosaccharides are poorly recognized. The majority of VAO-type oxidases perform alcohol oxidations (EC 1.1.3.x) resulting in the production of aldehydes, ketones or lactones. Nonetheless, VAO, the prototype enzyme of this flavoprotein family, is not only able to perform alcohol oxidations but it is also capable to perform amine oxidations, hydroxylations and ether bond cleavage reactions [13]. Beside the aforementioned reactions, the VAO family includes also oxidases capable of C-C bound formation [14]. One known example is reticuline oxidase (also known as berberine bridge enzyme) involved in the biosynthetic pathway of plant isoquinoline alkaloids [15].

In an effort to discover novel oxidative biocatalysts acting towards biomass or biomass derived compounds, we specifically investigated the genome of the thermophilic fungus *M. thermophila*. This fungus is known to convert plant biomass and is a producer of a plethora of biomass converting enzymes. In addition, an effective *M. thermophila* expression system has been developed, enabling the production of high levels of recombinant proteins through homologous (and heterologous) expression. As *M. thermophila* is a thermophilic fungus, its proteins would withstand higher temperatures, making them optimal choices for industrial purposes.

We recently reported on the discovery and the elucidated crystal structure of a new VAO-type oxidase: xylooligosaccharide oxidase (XylO) from *M. thermophila* XylO has a similar active site when compared to previously determined structures of other known VAO-type carbohydrate oxidases such as glucooligosaccharide oxidase from *Acremonium strictum* (GOOX) and lactose oxidase from *Microdochium nivale* (LaO) [10]. Yet, for each of these enzymes, delicate differences in active site residues result in different substrate acceptance profiles. During the work that led to the discovery of XylO, other putative VAO-type oxidases were identified in the predicted proteome of *M. thermophila*. In this study we report on the characterization of two of these VAO-type flavoproteins.

## 2. Results

### 2.1. Identification of M. thermophila Genes Encoding Putative VAO-Type Flavoprotein Oxidases

**Figure 1:**
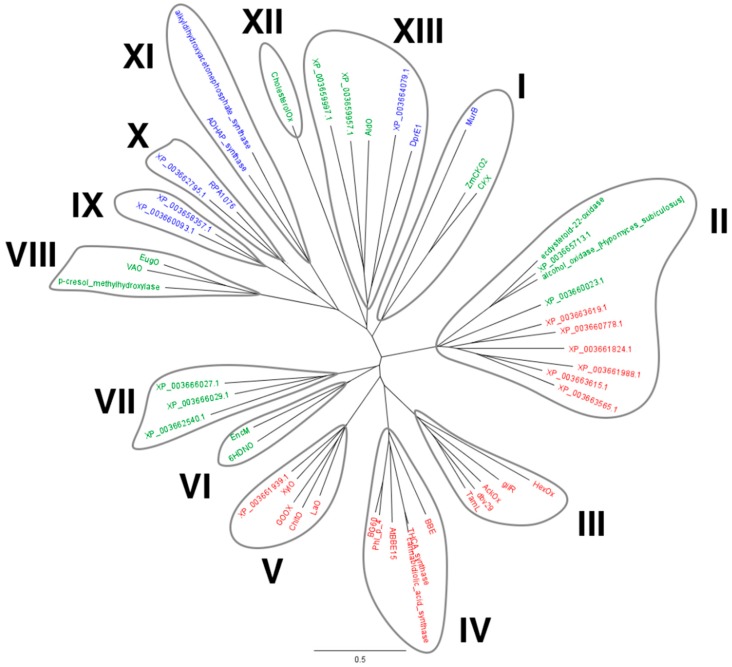
Phylogenetic tree of the VAO-type flavoproteins in the genome of *M. thermophila* supplemented with sequences of VAO-type proteins for which structures have been determined. Some covalent flavoproteins for which a structure is not available are also included: alcohol oxidase from *H. subiculosus* (ACD39759), ecdysteroid-22-oxidase from *N. rileyi* (BAM11133), and ChitO from *Fusarium graminearum* (XP_011325372). HexOx: hexose oxidase from *Chondrus crispus* (AAB49376), GilR: oxidoreductase from *Streptomyces griseoflavus* (Q7X2G7), AckOx: aclacinomycin oxidoreductase from *Streptomyces galilaeus* (ABI15166), dbv29: oxidase from *Nonomuraea* sp. ATCC 39727 (CAD91224), TamL: oxidase from *Streptomyces* sp. 307-9 (ADC79636), BBE: reticuline oxidase from *Eschscholzia californica* (P30986), THCA-synthase: Δ1-tetrahydrocannabinolic acid synthase from *Cannabis sativa* (Q8GTB6), cannabidiolic_acid_synthase: cannabidiolic acid synthase from *C. sativa* (AKC34419), Phl_p_4 from *Phleum pretense* (P43213), BG60 from *Cynodon dactylon* (AAS02108), LaO: carbohydrate oxidase from *M. nivale* (3RJA), GOOX from *A. strictum* (2AXR), XylO from *M. thermophila* (XP_003663758), MurB: MurB from *Pseudomonas aeruginosa* (4JAY), PCMH: p-cresol methylhydroxylase from *Pseudomonas putida* (P09788), VAO: vanillyl alcohol oxidase from *Penicillium simplicissimum* (P56216), EugO: eugenol oxidase from *Rhodococcus jostii* (5FXD), AtBBE15 from *Arabidopsis thaliana* (4UD8), HDNO: 6-hydroxy-d-nicotine oxidase from *Arthrobacter oxydans* (P08159), EncM: EncM from *Streptomyces maritimus* (Q9KHK2), CholesterolOx: cholesterol oxidase from *Brevibacterium sterolicum* (1I19), AldO: alditol oxidase from *Streptomyces coelicolor* (2VFR), DprE1: decaprenylphosphoryl-beta-d-ribose oxidase from *M. tuberculosis* (4FDN), ADHAP_synthase: alkyldihydroxyacetonephosphate synthase from *Cavia porcellus* (4BBY), alkyldihydroxyacetonephosphate synthase from *Dictyostelium discoideum* (2UUU), RPA1076: a putative dehydrogenase from *Rhodopseudomonas palustris* CGA009 (3PM9), ZmCKO2: cytokinin oxidase/dehydrogenase 2 from *Zea mays* (4ML8), CKX: cytokinin oxidase/dehydrogenase from *Arabidopsis thaliana* AT5G21482 (2EXR). The phylogenetic tree was generated with Geneious, version 7.0 (http://www.geneious.com, Biomatters, Silkeborg, DK). In red: bicovalent flavoproteins. In green: monocovalent flavoproteins. In blue: non covalent flavoproteins.

Using the sequence of the bicovalent flavoprotein ChitO as query, a BLAST search in the predicted proteome of *M. thermophila* was performed. Nineteen putative VAO-type flavoproteins were identified and subsequently aligned together with characterized VAO-type flavoproteins. Upon multiple sequence alignment of the recovered protein sequences with sequences from VAO-type flavoproteins for which a crystal structure was elucidated, a phylogenetic tree was built (Figure 1). Given their high similarity with MtVAO713, two additional sequences were included in the tree: an alcohol oxidase from *Hypomyces subiculosus* (accession number: ACD39759) and ecdysteroid-22-oxidase from *Nomuraea rileyi* (accession number: BAM11133). Thirteen clades can be identified in this phylogenetic tree. Eight clades mainly consist of known flavoproteins (clades I, III, IV, V, VI, VIII, XI, and XII). Clade V consists of the characterized carbohydrate oxidases, which includes XylO, while also another homolog (XP_003661939.1) of *M. thermophila* is part of this group. This suggests that *M. thermophila* contains another carbohydrate oxidase. Other VAO-type proteins from *M. thermophila* are found in clades I, II, V, VII, IX, X, and XIII. Through SignalP (www.cbs.dtu.dk/services/SignalP), eleven proteins of *M. thermophila* were predicted to contain a signal sequence for secretion: XylO, XP_003661939, XP_003663615, XP_003663619, XP_003662540, XP_003661824, XP_003663565, XP_003660778, XP_003665713, XP_003660023, XP_003659997. Interestingly the majority of these proteins are predicted to be bicovalently bound while only four (XP_003662540, XP_003665713, XP_003660023, XP_003659997) are predicted to be monocovalently bound. We selected two flavoproteins for further studies: XP_003663615 (MtVAO615), a putative bicovalent flavoprotein, and XP_003665713 (MtVAO713), a putative monocovalent flavoprotein. Interestingly, MtVAO713 has a Ser at the position where normally a Cys is present in bicovalent flavoproteins, forming the Cys-FAD linkage. Both proteins are part of a group of putative oxidases (clade II, see Figure 1) for which hardly any biochemical data are available and we set out to explore their catalytic and structural properties.

### 2.2. Purification

**Figure 2:**
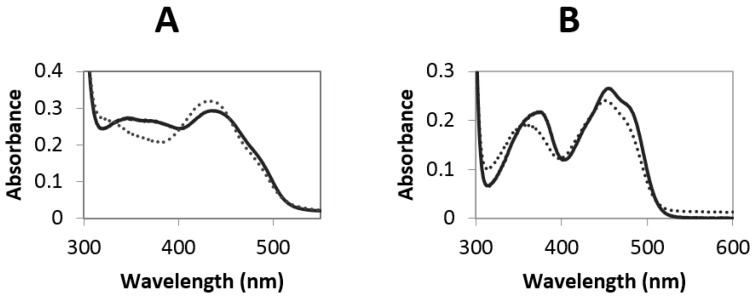
(**A**) Spectrum of 26 µM native MtVAO615 (solid line) and unfolded MtVAO615 upon addition of 0.1% SDS and heating the sample at 80 °C for 5 min (dashed line), in 20 mM Tris/Cl pH 7.6; (**B**) Spectrum of 20 µM native MtVAO713 (solid line) and unfolded MtVAO713 upon addition of 0.1% SDS and heating the sample at 80 °C for 5 min (dashed line), in 20 mM Tris/Cl pH 7.6.

From 50 mL of concentrated fermentation supernatant, around 20 mg of MtVAO615 was obtained after two purification steps. The purified protein displays a bright yellow color and exhibits and absorbance spectrum with maxima at 349 and 446 nm (Figure 2A). The spectral features (the 349 nm peak is rather broad and the 446 nm peak has a pronounced should at around 480 nm) are reminiscent of those of bicovalent flavoproteins. The purified protein runs in SDS-PAGE as a single band at around 70 kDa while the expected size is predicted to be 60 kDa. This indicates the presence of post-translational modifications such as glycosylation. Upon incubation of the gel with 5% acetic acid, the protein displayed clear fluorescence, confirming the presence of covalently bound FAD.

From 50 mL of concentrated fermentation supernatant, around 45 mg of MtVAO713 was obtained after two purification steps. The purified protein showed a bright yellow color and its absorbance spectrum displays maxima at 372 and 454 nm (Figure 2B). The absorbance spectrum is remarkably different from the one of MtVAO615 and hints to a non- or monocovalently bound flavin cofactor. On SDS-PAGE the purified fraction runs as a single band at around 60 kDa which is the expected size for this protein. This suggests that this protein is not or not significantly glycosylated. Similar to MtVAO615, the protein displayed clear fluorescence upon SDS-PAGE and acetic acid treatment, indicating the presence of covalently bound FAD.

### 2.3. Redox Potential Determination and Stopped Flow Experiments

The redox potential of the FAD in both enzymes was investigated by using the xanthine oxidase-based method [16]. Using this method, a full reduction of both enzymes could be observed with no significant formation of a radical species. We were able to determine a redox potential of +23 mV for MtVAO713 using methylene blue (+11 mV) as reference dye (Figure S2). Such a relatively high redox potential is typical for monocovalent flavoproteins. For MtVAO615 we were unable to calculate the redox potential since the redox potentials of the tested dyes were too far off from the one of the enzyme. In the redox titrations MtVAO615 became nearly fully reduced before thionin acetate (+64 mV) was reduced (Figure S3) while with 2,6-dichloro indophenol (+217 mV) it was the other way around. No other suitable reference dyes could be obtained. These observations indicate that the redox potential of MtVAO615 is extremely high for a flavoprotein, around 120–160 mV, which is close to the values reported for other bicovalent flavoproteins [5].

After having purified both proteins and having confirmed the presence of a covalent flavin cofactor, we set out to determine whether the proteins can function as oxidases. The typical characteristic of an oxidase is its capability to use molecular oxygen as electron acceptor. To test if the two purified proteins are oxidases, we first reduced anaerobically their flavin cofactor with sodium dithionite. Reduction could be witnessed by the disappearance of the yellow color of the oxidized flavin. After full reduction of the proteins, the proteins were mixed with a buffer containing dioxygen. By using a stopped-flow machine, the reappearance of oxidized flavin could be monitored in millisecond time scale. When mixing the reduced proteins with dioxygen, in both cases a rapid reoxidation of the reduced flavin was observed. Kinetic analysis of the spectral scans revealed that the reoxidation occurs in two (MtVAO713) or three (MtVAO615) steps (Figures S4 and S5). In both cases reappearance of the fully oxidized flavin spectrum mainly occurs in the first fast process with rates of 14.2 ± 0.1 s^−1^ and 75.0 ± 0.1 s^−1^, respectively. The subsequent kinetic events resulted in minor spectral changes which may reflect formation of flavin radical species or damaged flavin cofactor species, due to the intensity of the light source used for diode array detection. Therefore, they are probably irrelevant kinetic events. As a solution of dioxygen saturated buffer was used for reoxidation, the bimolecular rate constants could be calculated: 2.4 × 10^4^ M^−1^ s^−1^ for MtVAO713 and 1.3 × 10^5^ M^−1^ s^−1^ for MtVAO615. These rates are in the same range as those of known flavoprotein oxidases [17]. When taking into account that the rate of reoxidation is often even enhanced when product is still bound to the active site, these data show that both proteins are true flavoprotein oxidases.

### 2.4. Substrate Screening

Once the oxidase activity was confirmed for both flavoproteins, we set out to explore their substrate scope. First, a panel of twenty-three carbohydrates (see Materials and Methods was tested using the HRP-based assay. This assay couples the production of hydrogen peroxide, which is formed upon substrate oxidation by an oxidase, with the HRP-catalyzed formation of a purple product. Unfortunately, MtVAO615 nor MtVAO713 showed any activity towards the carbohydrates tested.

As can be seen in the phylogenetic tree (Figure 1), MtVAO713 is relatively closely related to an alcohol oxidase from *H. subiculosus* (GeneBank accession number: ACD39759.1; 59% sequence identity) [18] and the ecdysteroid-22-oxidase from *N. rileyi* (GeneBank accession number: BAM11133.1; 52% sequence identity) [19]. Both fungal enzymes are acting on rather bulky secondary alcohols. Hinted by this, a plethora of steroids and secondary alcohols were tested by either the HRP assay or GC/MS in order to identify potential substrates. This revealed one substrate for MtVAO713: ricinoleic acid. Ricinoleic acid is the major component of the castor oil which is obtained from the pressing of the seeds of the plant *Ricinus communis*. It is an unsaturated omega-9 fatty acid hydroxylated in position 12. The oxidation in position 12 results in the formation of 12-ketooleic acid. Recently, a Korean group reported on the successful whole cell conversion of ricinoleic acid to 12-ketooleic acid by using a recombinant strain of *Corynebacterium glutamicum* expressing a secondary alcohol dehydrogenase from *Micrococcus luteus* [20]. The conversion of ricinoleic acid by MtVAO713 was confirmed by GC/MS analysis (Figures S6 and S7). Formation of 12-ketooleic acid could be measured. Nevertheless, the conversion rate is very poor as only 0.5% of 5 mM ricinoleic acid was converted in 16 h by 8 μM of MtVAO713. This indicates that ricinoleic acid is not the optimal substrate for this enzyme. Yet, it confirms that MtVAO713 can act as an alcohol oxidase and that it can convert secondary alcohols, a rare oxidase activity. Several ricinoleic acid derivatives were tested to unravel which functional groups are important for activity. None of the tested related compounds (methyl ricinoleate, ricinoleyl alcohol, 12-hydroxystearic acid, 12-hydroxydodecanoic acid) was found to be converted as measured by GC/MS.

For MtVAO615 a similar approach did not bring any lead. When examining the phylogenetic tree, no close homologs could be identified for which any biochemical data are available. A BLAST search results in several homologs with putative functions which have not been characterized yet. Several steroids, secondary alcohols and other different molecules were tested as potential substrate but no activity could so far be identified. Also, complex mixtures of substrates were tested as ground rye, oats, wheat, barley, wheat straw, arabinan and wheat arabinoxylan. These substrates would provide not only oligo- and polysaccharides but also other components of the plant material. MtVAO615 did not show activity with these complex mixtures.

### 2.5. Crystal Structures

To have a better insight into the properties of both oxidases, their crystal structures were determined (Table 1). The structures of MtVAO615 and MtVAO713 were both determined by molecular replacement. The final model of MtVAO713 comprises 4 protein molecules with amino acid residues 27–598 and 4 FAD molecules. Electron density maps show the presence of *N*-glycosylation at Asn103 and Asn131. The final R-factors are 19.8/23.9 (*R_cryst_*/*R_free_*). The crystal structure of MtVAO615 with amino acid residues 27–574 was solved in two different space groups. The structure at pH 7.5 contains one monomer while the structure at pH 5.0 contains two monomers. *Rcryst*/*Rfree* are 19.5/23.5 and 23.6/28.0 respectively. As already observed by SDS-PAGE, MtVAO615 is heavily glycosylated, showing electron density in 2F_o_-F_c_ maps for *N*-glycosylation at Asn47, Asn105, Asn129, Asn211, Asn310 and Asn438.

Both proteins belong to the VAO flavoprotein family. VAO-type proteins are composed of a FAD-binding domain and substrate domain [21]. MtVAO615 and MtVAO713 have 29% sequence identity and have an rmsd of 2.0 Å on 515 Cα atoms. The structural differences are mainly situated in loops on the exterior of the proteins. Sequence alignment indicated that MtVAO615 and MtVAO713 contained an extra N-terminal domain of about 100 amino acid residues compared to other VAO flavoproteins. However, the extension is only ~10 residues. The remainder of the extra residues is found in 2 large loops in the FAD binding domain, several smaller loops and the C-terminal extension of ~10 amino acid residues (Figure S1). The first 26 residues of both proteins are part of secretion signal sequences. The MtVAO615 and MtVAO713 structures are similar to other structurally characterized VAO family members. The most similar structures to MtVAO615 are that of a flavoenzyme from *Streptomyces maritimus* (EncM, PDB code 3W8W, 22% sequence identity rmsd 2.2 Å) [22], 6-hydroxy-d-nicotine oxidase from *Arthrobacter nicotinovorans* (6HDNO, PDB code 2BVF, 20% sequence identity, rmsd 2.3 Å [23]), aclacinomycin oxidoreductase from *Streptomyces galilaeus* (AckOx, PDB code 2IPI, 22% sequence identity rmsd 2.4 Å [8]), tirandamycin oxidase from *Streptomyces* sp. 307-9 (TamL, PDB code 2Y08, 22% sequence identity, rmsd 2.3 Å [9]) and lactose oxidase from *Microdochium nivale* (LaO, PDB code 3RJ8, 21% sequence identity, rmsd 2.1 Å, to be published). For MtVAO713 these values are 21%, 2.6 Å (EncM); 20%, 2.8 Å (6HDNO); 15%, 2.6 Å (AckOx); 17%, 2.4 Å (TamL); and 20%, 2.4 Å (LaO). Structural similarity to MtVAO713 was highest for glucooligosaccharide oxidase from *Acremonium strictum* (GOOX, PDB code 1ZR6 19% seq. id., rmsd 2.3 Å [4]) and an oxidoreductase from *Streptomyces griseoflavus* (GilR, PDB code 3POP, 18% seq. id., rmsd 2.5 Å [24]). The FAD domains of both structures are well conserved while conservation in the S domains is much less, accounting for diverse substrate specificities (Figure S1).

Four disulfide bridges are present in MtVAO615 while in MtVAO713 six disulfide bridges exist. In both MtVAO615 and MtVAO713 the cofactor FAD is covalently tethered to the protein by covalent linkages. As expected, MtVAO615 harbors a bicovalently linked 6-*S*-cysteinyl-8α-*N*1-histidyl FAD while MtVAO713 has a monocovalently linked 8α-methyl-*N*1-histidyl FAD (Figure S1). The isoalloxazine ring of the cofactor is covalently linked with the C6 atom to the Sγ of Cys222 and with the 8α-methyl group to the Nδ1 of His157 in MtVAO615 and His159 in MtVAO713. Cys222 in MtVAO615 is substituted for Ser231 in MtVAO713. All structural homologs have a covalently 8α-methyl-*N*1-histidyl FAD while at the Cys222/Ser231 position, also Val or His residues are found (Figure S1).

**Figure 3:**
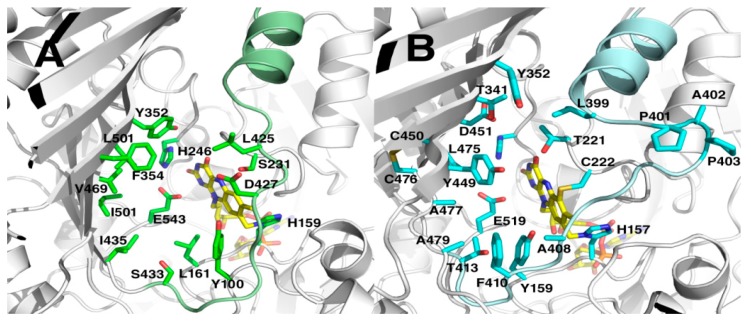
View of the predicted active sites of MtVAO713 (**A**) and MtVAO615 (**B**). The bound FAD (yellow) and active site pocket residues are in stick representations.

The entrances to the active sites are shaped by the seven-stranded β-sheet of the substrate domain and α-helix11 with a loop (res. 391–413 for MtVAO615, res. 415–433 for MtVAO713). The groove shaped substrate binding pocket of MtVAO615 is solvent accessible and contains, besides His157 and Cys222 which tether the FAD, Tyr159, Thr221, His237, Thr341, Tyr352, Leu399, cis-Pro401, Ala402, cis-Pro403, Ala408, Phe410, Thr413, Tyr449, Asp451, Leu475 Ala477, Ala479, Glu519 (Figure 3B). The structure of the loop is formed by two cis-prolines and is probably important for substrate binding. On the other side of the active site the disulfide Cys450-Cys476 stabilizes β-strands 17 and 18 with residues lining the active site. The groove to the substrate binding pocket of MtVAO713 is broader than of MtVAO615 and is delimited by Tyr100, Leu161, Ser231, His246, Tyr352, Phe354, Leu425, Asp427, Ile435, Ser433, Val469, Leu471, Ile501 and Glu543 (Figure 3A). The active site opposite to the FAD is very hydrophobic and is lined by several leucines and isoleucines. The hydrophobic nature of these residues is in line with the identified substrate ricinoleic acid, which is a very hydrophobic compound.

Among all the structurally characterized bicovalent flavoproteins, a common feature is the wide opening of the active site on the surface. This contrasts with monocovalent or noncovalent flavoproteins in which the active site is more buried. This feature allows bicovalent flavoproteins to accommodate bulky substrates in their active site [5]. For instance, carbohydrate oxidases in which the FAD is bicovalently bound accept oligosaccharides with higher efficiency compared to monosaccharides [3,11,12]. Protein MtVAO713 and MtVAO615 are both an exception to this rule. Despite having a monocovalent FAD, the active site of MtVAO713 is rather open, similar to bicovalent flavoproteins (Figure 3A). MtVAO713 is also closely related to other bicovalent flavoproteins concerning its sequence and structure. This suggests that MtVAO713 has evolved from a bicovalent flavoprotein.

On the other hand, despite having a bicovalently bound FAD cofactor, access to the active site of MtVAO615 is rather narrow (Figure 3B). In particular, Phe410 and Tyr449 seem to create a sort of funnel. It is worth to note that Phe410 is on a loop which runs longitudinally across the active site. While this loop is further away from the active site in the other sequence-related oxidases, in MtVAO615 it leans towards the active site contributing to narrowing the cavity. In physiological conditions and in the presence of the native substrate, the loop might be mobile to allow substrate binding.

When inspecting the active site residues of both MtVAO615 and MtVAO713, striking differences between these two proteins and the known structurally resolved oxidases can be noted. Table 2 lists the active site residues of various VAO-type flavoproteins for which a crystal structure is available. This shows that, while MtVAO615 and MtVAO713 have a strikingly similar active architecture, their active site residues are very different from other VAO-type oxidases. For example, a structurally conserved Tyr often assists in catalysis in most VAO-type oxidases which is missing in MtVAO615 and MtVAO713: a Glu residue is present at this position, respectively Glu519 and Glu543 (Table 2). It has been proposed that Tyr429 in GOOX acts as a base which abstracts a proton from the O1 of the substrate, contributing to the hydride transfer to FAD. A similar catalytic role is played by Tyr473 in Dbv29 [7], Tyr448 in GilR [24], Tyr447 in TamL [9], Tyr450 in aclacinomycin oxidoreductase (AckOx) [8] and Tyr484 in THCA synthase [25] (position ‘519’ in Table 2). Asp355 in GOOX may assist in the proton transfer by lowering the pKa value of Tyr429 through a water molecule [4]. MtVAO615 has an Asp at a similar position, while MtVAO713 has a Leu (position 451 in Table 2). When comparing the active site residues, it seems that MtVAO615 and MtVAO713 represent a new subclass of oxidases with a totally new constellation of active site residues. While we could confirm oxidase activity on a secondary alcohol for MtVAO713, the real substrates for these oxidases still need to be identified. With the unique active sites, the oxidases may even catalyze unprecedented oxidative reactions as other sequence-related VAO-type oxidases have been shown to display various exotic oxidative reactions which include ether bond cleavage and C-C bond formation reactions.

Inspection of the structures of both oxidases revealed a feature that is in line with the efficient oxygen reactivity. Recently a so-called gatekeeper residue which tunes oxygen reactivity of VAO-type flavoproteins was identified on the re-side of the isoalloxazine ring [26]. While this residue is typically rather bulky in homologs that are poorly reactive with dioxygen, in these newly identified oxidases residues (Val225 in MtVAO615 and Pro234 in MtVAO713) are found that provide enough space for dioxygen to reach and react with the reduced flavin cofactor. Taken together, the kinetic and structural data indicate that the two studied flavoproteins are bona vide oxidases.

## 3. Discussion

In the present study we report the discovery and the structural characterization of two novel flavoprotein oxidases from the thermophilic fungus *M. thermophila*. Both present rather unique structural features in the active site. Only for MtVAO713 we could identify ricinoleic acid as substrate (although a very poor one) which paves the way for further exploration.

The elucidation of the crystal structures can be used to rational design a library of compounds to be virtually screened through automated docking. Unfortunately this approach suffers of several limitations: (1) when lacking a lead compound, the creation of the library of test compounds might be extremely challenging and time consuming; (2) the results of a docking procedure often only predict whether binding of the compound is likely while it does not guarantee whether the binding pose will be productive, enabling catalysis; (3) the current docking procedures lacks accuracy in predicting binding energies which is partly due to the fact that typically the enzyme structure is taken as a rigid configuration.

Another approach for identifying a substrate for a newly found enzyme is testing complex mixtures of natural sources, for example extracts of plant biomass can be used to screen by chemical analysis for chemical modifications. This approach is less specific but in this lies its advantage when no indication whatsoever is available as to which substrate would work with these novel enzymes. In particular for MtVAO713, extracts can be generated from flax and/or oats hulls and be tested with the enzyme. The analysis will present definitely challenges given the high heterogeneity of the sample. Pre-fractionation of the extract could be used to reduce the sample complexity. Alternatively, it may be tempting to use protein crystals to soak them in a (complex) mixture of test compounds. When this is performed anaerobically, substrate will bind and by structure elucidation, the identity of tight binding substrates will be revealed.

## 4. Materials and Methods

### 4.1. Bioinformatic Analysis and Expression

The sequence of ChitO (accession number: XP_011325372) was used as query to perform a protein BLAST search on the “Non-redundant protein sequences” database with *M. thermophila* ATCC 42464 (taxid: 573729) set as organism (https://blast.ncbi.nlm.nih.gov/Blast.cgi). Protein sequences were exported in the FASTA format and aligned with the software Geneious using default parameters. From the multiple sequence alignment, a phylogenetic tree was obtained with the following parameters: Genetic distance model: Jukes Cantor; Tree build method: Neighbor-Joining; Outgroup: No outgroup. Cloning and expression of MtVAO713 and MtVAO615 were performed as described in [10].

### 4.2. Purification

Purification was performed similar to a procedure of a previous study [10]. 50 mL processed fermentation broths for each protein were received from Dupont frozen at −20 °C. Samples were thawed on ice and buffer exchanged with 20 mM Tris/Cl pH 7.6. Subsequently they were loaded on pre-equilibrated 5 mL Hi-Trap Q-sepharose column using a FPLC purification system (ÅKTA Purifier) (GE Healthcare Europe GmbH, Eindhoven, NL). Absorbance was monitored at 280 nm, 445 nm and 600 nm. The column was washed until absorbance at 280 nm reached baseline levels (≈10 column volumes). Both proteins were eluted at 10% of elution buffer (20 mM Tris/Cl pH 7.6 and 2 M NaCl).

Yellow fractions which displayed absorbance at 445 nm were collected, concentrated and subsequently loaded on a Superdex 200 column. 50 mM acetate buffer pH 5.6 containing 100 mM NaCl was used to equilibrate the column and for the elution. The yellow fractions displaying absorbance at 445 nm were collected and concentrated.

### 4.3. Crystallization, Data Collection and Structure Determination of MtVAO615

Initial vapour-diffusion crystallization experiments were performed using a Mosquito crystallization robot (TTP Labtech, Hertfordshire, UK). In a typical experiment, 0.1 μL screening solution was added to 0.1 μL MtVAO615 protein solution (9.6 mg mL^−1^) on a 96-well MRC2 plate (Molecular Dimensions, Suffolk, UK); reservoir wells contained 50 μL screening solution. The screening solutions used for the experiments were PACT and JCSG+ (Molecular Dimensions). MtVAO615 crystals appeared after 3–30 d of incubation at 294 K in solutions containing PEG at pH 5 to 9. Crystallization conditions were optimized using hanging-drop set-ups containing 1 μL protein solution and 1 μL reservoir solution at 294 K. Crystals grown from 11% PEG3350, 20% glycerol and 0.1 M Hepes pH 7.5 and from 20% PEG6000, 0.2 M NaCl and 0.1 M sodium acetate at pH 5.0 were used for crystal structure determination.

Before data collection, crystals were briefly soaked in a cryoprotectant solution, consisting of 20% glycerol, 25% PEG3350 and 0.1 M Hepes pH 7.5. X-ray diffraction data to 2.0 Å resolution were collected from a single cryo-cooled crystal mounted on an in-house MarDTB Goniostat System using Cu-Kα radiation from a Bruker MicrostarH rotating-anode generator equipped with HeliosMX mirrors. Intensity data were processed using iMosflm [27]. The diffraction patterns were anisotropic with a weak zone of decreased resolution (2.7 Å) resulting in poor statistics for the high resolution shell (Table 1). The crystals belong to spacegroup *P*212121 with one monomer of 54 kDa in the asymmetric unit. The VM is 2.7 Å^3^/Da [28] with a solvent content of 54%. Crystals grown in one month at pH 5.0 were also cryocooled with 20% glycerol. They belonged to space group P22121 and diffracted to 2.2 Å resolution. With two monomers in the asymmetric unit the solvent content is 57% and V_M_ is 2.9 Å^3^/Da [28]. Intensity data were processed with XDS [29]. Data collection statistics are listed in Table 1.

Five structures were selected with highest identity to the C-terminal part of MtVAO615 i.e., 6HDNO (PDB code: 2BVF) [23], AckOx (PDB code: 2IPI) [8], TamL (PDB code: 2Y08) [9], EncM (PDB code: 3W8W) [22], and LaO, (PDB code: 3RJ8). No homology model could be obtained for the 100 N-terminal amino acids because of lack of sequence homology. Using these five structures as templates, homology models were generated with the SCWRL server [30]. Molecular replacement was performed with the program Phaser [31] using an ensemble of the five homology models. 

Although Phaser was able to find a clear solution for the *P*2_1_2_1_2_1_ data with one monomer in the asymmetric unit, the calculated initial phases were not good enough to build the structure. This is probably caused by the low sequence identity and the anisotropically diffracting crystals. The phases calculated by Phaser for two monomers in the asymmetric unit in the P22_1_2_1_ data were sufficient for automatic model building by the Phase and build program in the PHENIX suite [32]. The refined model was used in determining the structure in the *P*2_1_2_1_2_1_ space group by Phaser. 

### 4.4. Crystallization, Data Collection and Structure Determination of MtVAO713

Before crystallization of MtVAO713, an extra step of enzyme purification (and buffer exchange) was performed using a Superdex200 10/300 GL column (GE Healthcare Europe GmbH, Eindhoven, NL), equilibrated with 20 mM Hepes buffer, pH 7.5, containing 150 mM NaCl. MtVAO713 eluted at a molecular weight of 53 kDa. Only the first fractions of the MtVAO713 peak containing FAD were pooled and concentrated. Dynamic light scattering (DLS) analysis (DynaPro NanoStar, Wyatt technology, Santa Barbara, CA, USA) indicates that the protein has a hydrodynamic radius of 3.38 nm, with an apparent molecular weight of 58 kDa and 5% polydispersity.

Crystallization conditions were screened using the Mosquito crystallization robot with a MtVAO713 solution of 10–23 mg mL^−1^ (see above). Many commercially available screens were tried but only with the Morpheus screen (Molecular Dimensions) thin yellow plate-like crystals appeared after 1 month of incubation at 294 K. Conditions were optimized using hanging-drop set-ups with 25% PEG3350 and 0.1 M Tris pH 8.5–9.0 as precipitant, and drops containing 1 μL protein solution (10 mg mL^−1^) and 1 μL reservoir solution.

Crystals were soaked in a cryoprotectant prepared by the addition of 20% (*w*/*v*) glycerol to the reservoir solution. X-ray diffraction data to 2.2 Å resolution were collected from a single cryo-cooled crystal mounted on the in-house X-ray source. Intensity data were processed using XDS [29]. The crystals (apparently) belong to space group P22121 with *a* = 83.2 Å, *b* = 108.5 Å and *c* = 136.0 Å and all angles 90°. With two monomers of 58 kDa in the asymmetric unit the VM is 2.4 Å^3^/Da [28] with a solvent content of 48%.

MtVAO713 has 24% identity to MtVAO615 and this structure was used as a template for molecular replacement. The homology model was generated with the SCWRL server [30] and molecular replacement was performed with the program Phaser [31]. Phaser obtained a distinct solution for the first monomer but a solution for the second monomer could not be calculated. Analysis of the data revealed systematic absences for (0, k, 0) with k odd and for (0, 0, l) with l odd. However, the 00l reflections only spike at intermediate resolution displaying l = 2n presence, breaking down on lower and higher resolution suggesting a lower symmetry space group. After processing the data in space group P21, with a β angle of 90.00, Phaser was able to find four molecules in the asymmetric unit. Molecules A/D and B/C are related by an NCS operator that is close to a perfect twofold crystallographic rotation having P222 pseudosymmetry. The phases were good enough to build a model automatically with the Phase and build program in the PHENIX suite [32]. Data collection statistics are listed in Table 1.

### 4.5. Refinement

REFMAC5 was used for refinement of the models [33] and Coot [34] was used for manual rebuilding and map inspection. In the last rounds of refinement TLS groups were used. The quality of the models was analyzed with MolProbity [35], secondary structure elements were assigned with DSSP [36] and Promals3D was used for structure alignment [37]. Figures were prepared with PyMOL (http://www/pymol.org) and ESPript [38]. Atomic coordinates and experimental structure factor amplitudes have been deposited in the Protein Data Bank (PDB) with PDB IDs 6F72, and 6F73 and 6F74 for monomeric, dimeric MtVAO615 and MtVAO713, respectively.

### 4.6. Carbohydrates and Primary Alcohol Substrate Screening

The screening was performed as previously described [39]. In brief, oxidase activity of the two enzymes was detected by coupling H_2_O_2_ production to the oxidation of 4-aminoantipyrine and 3,5-dichloro-2-hydroxybenzenesulfonic acid by horseradish peroxidase (HRP). The formation of the resulting pink/purple colored product can be followed at 515 nm (ε_515_ = 26 mM^−1^ cm^−1^). The reaction mixture for the carbohydrates/primary alcohol substrate screening contained 50 mM phosphate buffer, pH 7.6, 0.1 mM 4-aminoantipyrine, 1.0 mM 3,5-dichloro-2-hydroxybenzenesulfonic acid, 4.0 units HRP and 1 μM of either enzymes. A panel of different carbohydrates comprising monomeric, dimeric, tetrameric, polymeric, cyclooligosaccharides and a range of primary alcohols were tested (see below). Potential substrates were tested in duplicate using concentrations of 50 mM and 5.0 mM for mono- and disaccharides, and 1.0 mM and 0.5 mM for tetra- and cyclo-oligosaccharides. To exclude pH effect, xylobiose and cellobiose in 50 mM and 5.0 mM were tested in pH range from 4.0 to pH 9.0 using the Britton&Robinson buffer. For screening complex mixtures 2 μM of either enzyme were used to detect oxidase activity with the HRP assay on complex mixtures of substrates. The assay was performed in 50 mM acetate buffer pH 5.6 on 50 mg/mL ground rye, oats, wheat, barley, wheat straw, arabinan and wheat arabinoxylan. Full list of tested compounds: for MtVAO713: glucose, galactose, fructose, mannose, xylose, arabinose, *N*-acetyl-d-glucosamine, sucrose, maltose, lactose, sorbitol, xylitol, cellobiose, cellotetraose, α-cyclodextrin, β-cyclodextrin, arabinan, chitosan, chitin, starch, maltotetraose, glycerol, hydroxymethylfurfural, xylobiose, testosterone, cholesterol, beta-ecdysone, 20-hydroxyecdysone, ergosterol, cholecalciferol, beta-sitosterol, stigmasterol, ricinoleic acid, methly ricinoleate, ricinoleyl alcohol, pinellic acid, cyclohexanol, 2-propanol, 2-butanol, 2-pentanol, 2-heptanol, 1-amino-2-propanol, 3-pentanol, 3-heptanol, 3-octanol; for MtVAO615: glucose, galactose, fructose, mannose, xylose, arabinose, *N*-acetyl-d-glucosamine, sucrose, maltose, lactose, sorbitol, xylitol, cellobiose, cellotetraose, α-cyclodextrin, β-cyclodextrin, arabinan, chitosan, chitin, starch, maltotetraose, glycerol, hydroxymethylfurfural, xylobiose, ethanol, benzyl alcohol, glucose-6-phosphate, galactose-1-phosphate, mannose-6-phosphate, fructose-6-phosphate, xylan, arabinan, arabinoxylan, rye whole grain flour, oats flour, rye refined flour, wheat flour, barley flakes, straw, *N*-acetyl muramate.

### 4.7. Steroids and Secondary Alcohol Substrate Screening

1 μM of MtVAO713 was used to perform the conversion of either 1% or 5 mM of a panel of steroids and secondary alcohols (Supplementary information). As buffer 50 mM Tris/Cl pH 8.0 and 10% DMSO as co-solvent were used. Reactions were performed overnight at 30 °C or 37 °C at 300 RPM in a final volume of 1 mL in a 2 mL Eppendorf tube. Reactions were extracted twice with ethyl acetate and injected in the GC/MS. Ricinoleic acid and derived substrates i.e., methyl ricinoleate and ricinoleyl alcohol, were further processed for derivatization with the TMS reagent. Samples were first evaporated and then resuspended in 60 µL of MTBE (methyl tert-butyl ether). Subsequently 60 µL of 1% TMS (trimethylsilyl) in BSTFA (*N*,*O*-bis(trimethylsilyl) trifluoroacetamide) were added. This was followed by incubation at 75 °C for 30 min to allow the derivatization. The final samples were injected into GC/MS. The GC was performed on a HP1 column (dimethylpolysiloxan, 45 m × 0.25 mm × 0.25 µm) with a temperature gradient from 40 to 250 °C (5 °C/min) with a solvent cut-off of 6 min and a split ratio of 100. Ketooleic acid eluted at 36.9 min and ricinoleic acid at 37.2 min. 

### 4.8. Stopped Flow Analysis

The reoxidation of both enzymes was monitored by measuring the absorbance change of the FAD in presence of saturating O_2_ concentrations. A solution of approximately 40 μM of either enzyme was prepared in 50 mM phosphate buffer pH 7.6 to a final volume of 1 mL into a glass tube. The tube was flushed with N_2_ for 10–15 min. Then, sodium dithionite (10 mM) was added until the enzyme solution became bleached. In another glass tube a solution of only buffer was flushed for 10–15 min with O_2_. These solutions were mixed 1 to 1 in an Applied Photophysics SX20 stopped-flow apparatus and re-oxidation was monitored for 1 s using a photo diode array (PDA) detector between 186 and 724 nm. The spectral scans were fitted using equations for double or triple exponential decays.

### 4.9. Redox Potential Determination

The redox potentials were determined by using the method described by Massey [17]. Reactions were performed in 50 mM potatassium phosphate buffer pH 7.0 at 25 °C. A cuvette with MtVAO615 (10 μM) or MtVAO713 (5 μM), xanthine (400 μM), methyl viologen (2–5 μM) and redox dye (5–10 μM) was made anaerobically by flushing with N_2_ for 10–15 min. Subsequently, 50–60 μg of xanthine oxidase was added anaerobically to the cuvette and spectra were collected every 2 min during the reaction using a Jasco V-650 spectrophotometer (JASCO, Cremella, IT). The redox potentials were calculated by plotting the log ([ox]/[red]) of the protein versus log([ox]/[red]) of the redox dye according to Minnaert et al. [40]. As reference redox dyes we used methylene blue (E_M_ = +11 mV), thionin acetate (E_M_ = +64 mV) and 2,6-dichloro indophenol (E_M_ = +217 mV).

## Figures and Tables

**Table 1:** Data collection and refinement statistics. Values in parentheses are for the highest resolution shell.

	MtVAO615	MtVAO615	MtVAO713
Diffraction data	pH 7.5	pH 5.0	
Wavelength (Å)	1.54	1.54	1.54
Resolution range (Å)	51.4–2.0	58.0–2.2	54.3–2.2
Spacegroup	*P*2_1_2_1_2_1_	*P*22_1_2_1_	*P*2_1_
Cell dimensions (Å) *a*, *b*, *c*, β	59.7, 100.9, 111.8	62.4, 116.0, 198.7	83.2, 108.5, 136.0, 90.0
Number of unique reflections	45,720 (2754)	71,982 (4265)	120,924 (5895)
Completeness (%)	98.1 (81.1)	99.5 (92.8)	98.8 (97.3)
Overall I/σ (I)	4.7 (1.0)	12.9 (3.7)	3.9 (1.6)
*R_merge_* (%)	17.2 (98.6)	14.7 (51.4)	16.1 (48.3)
*R_pim_* (%)	11.4 (70.7)	5.8 (22.5)	14.7 (47.9)
*R/Rfree* (%)	23.6/28.0	19.5/23.5	19.8/23.9
R.m.s. deviations from ideal values			
Bond lengths (Å)	0.010	0.009	0.013
Bond angles (°)	1.444	1.333	1.575
Protein residues	27–475	27–475 (both molecules)	30–598 (4 molecules)
FAD molecule	1	2 × 1	4 × 1
Water molecules	366	850	1547
Glycerol molecules	-		8
NAG	9	4 (A), 7 (B)	4 × 3
β-mannose	1	-	-
PDB accession ID	6F72	6F73	6F74

**Table 2:** Amino acid positions relative to MtVAO615 in the active site of structurally charac-terized VAO-type flavoproteins (in grey hydrophobic residues, in red negatively charged residues, in blue positively charged residues, in green polar residues). Question marks indicate dubious residues given the lack of structural similarity with MtVAO615. 1DII: p-cresol methylhydroxylase from *P. putida*; 1VAO: VAO from *P. simplicissimum*; MtVAO713: XP_003665713.1 from *M. thermophila*; MtVAO615: XP_003663615.1 from *M. thermophila*; 3POP: GilR from *S. griseoflavus*; 2IPI: aclacinomycin oxidoreductase from *S. galilaeus*; 5AWV: Dbv29 from *N.* sp. ATCC 39727; 2Y08: TamL from *S.* sp. 307-9; 3D2D: reticuline oxidase from *E. californica*; 3VTE: Δ1-tetrahydrocannabinolic acid synthase from *C. sativa*; 4UD8: AtBBE15 from *A. thaliana*; 3TSH: Phlp4 from *P. pretense*; 4DNS: BG60 from *C. dactylon*; 3RJ8: lactose oxidase from *M. nivale*; 5L6F: XylO from *M. thermophila*; 2AXR: GOOX from *A. strictum*; ChitO from *F. graminearum*; 2BVF: 6-hydroxy-d-nicotine oxidase from *A. oxydans*; 3W8W: EncM from *S. maritimus*; 1I19: cholesterol oxidase from *B. sterolicum*; 2VFR: alditol oxidase from *S. coelicolor*; 4KW5: decaprenylphosphoryl-beta-d-ribose oxidase from *M. tuberculosis*; 4BBY: alkyldihydroxyacetonephosphate synthase from *C. porcellus*.

Position Relative to MtVAO615	159		237	451	475	477	519	
1DII	F		V	I	H	V	Y (?)	
1VAO	Y	S	V	T	H	I	Y (?)	
5FXD	G		V	I	H	V	Y (?)	
MtVAO713	L		H	L	V	I	E	
MtVAO615	Y		H	D	L	A	E	
3POP	G		Y	N	K	S	Y	
2IPI	F		Y	Y	K	W	Y	
5AWV	F		Y	I	K	N	Y	
2Y08	Y		Y	I	K	V	Y	
3D2D	Y		F	N	M	E	H	
3VTE	A		Y	Y	E	W	Y	
4UD8	Y		Y	N	K	Q	Y	
3TSH	Y		F	D	N	Q	Y	
4DNS	Y		F	D	N	Q	Y	
3RJ8	Y		F	D	L	Q	Y	
5L6F	Y		F	D	L	E	Y	
2AXR	Y		Y	D	L	Q	Y	
ChitO	Y		Y	D	L	Q	Y	
2BVF	P		V	E	E	L	N	
3W8W	M		F	L	V	N	F	
1I19	W		H	R	W	N	K	E
2VFR	F		H	R	A	H	K	
4KW5	Y		H	K	N	C	K	
4BBY	S		H	T	I	P	H (?)	

## Supplementary Materials

Supplementary materials are available online.
