# Supplementary material for: Characterization of Two VAO-Type Flavoprotein Oxidases from Myceliophthora thermophila
Source: Molecules. 2018 Jan 5;23(1):111. doi: 10.3390/molecules23010111 (PMC6017366; doi:10.3390/molecules23010111)
Supplement: Supplementary file 1 [file molecules-23-00111-s001.pdf]

Supplementary information

Characterization of two VAO-type flavoprotein oxidases from *Myceliophthora thermophile*

Alessandro R. Ferrari[1], Henriëtte J. Rozeboom[1], Aniek S.C. Vugts[2], Martijn J. Koetsier[2], Robert Floor[2], and Marco W. Fraaije[1,*]

[1] Molecular Enzymology, Groningen Biomolecular Sciences and Biotechnology Institute, University of Groningen, Groningen, The Netherlands
[2] Dupont Industrial Biosciences, Wageningen, The Netherlands
* Correspondence: m.w.fraaije@rug.nl; Tel: +31503634345

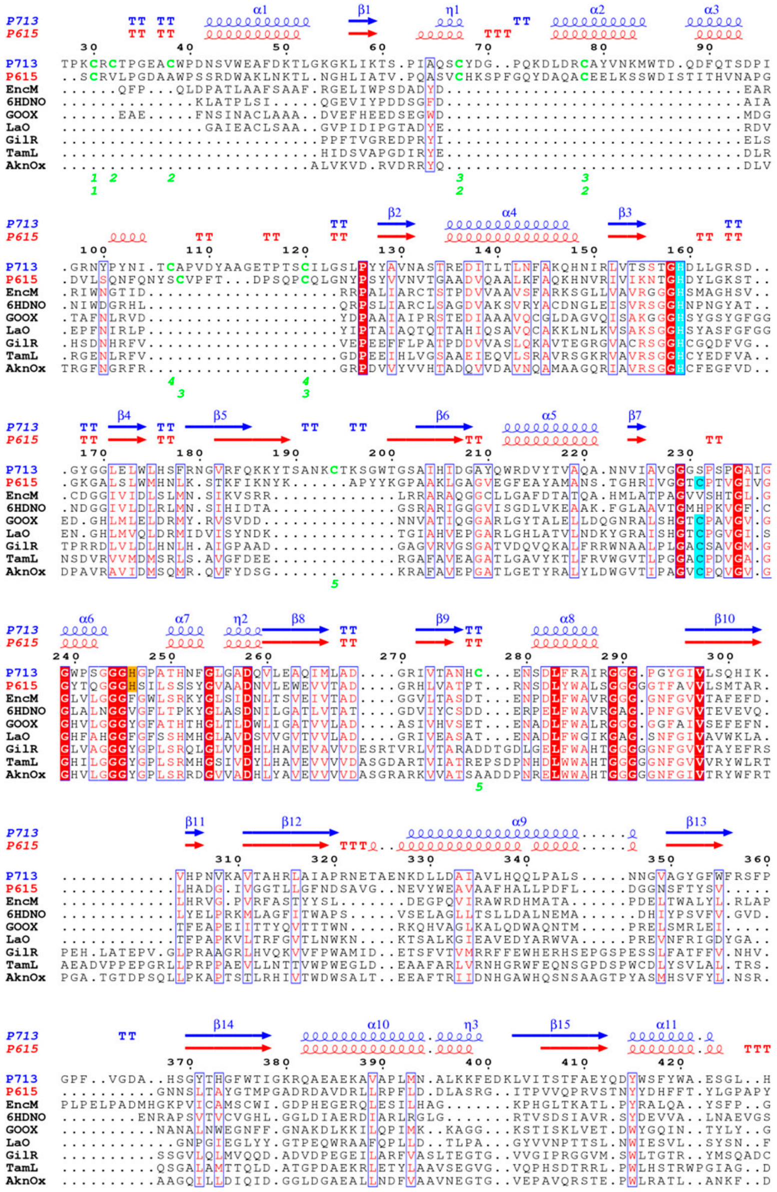

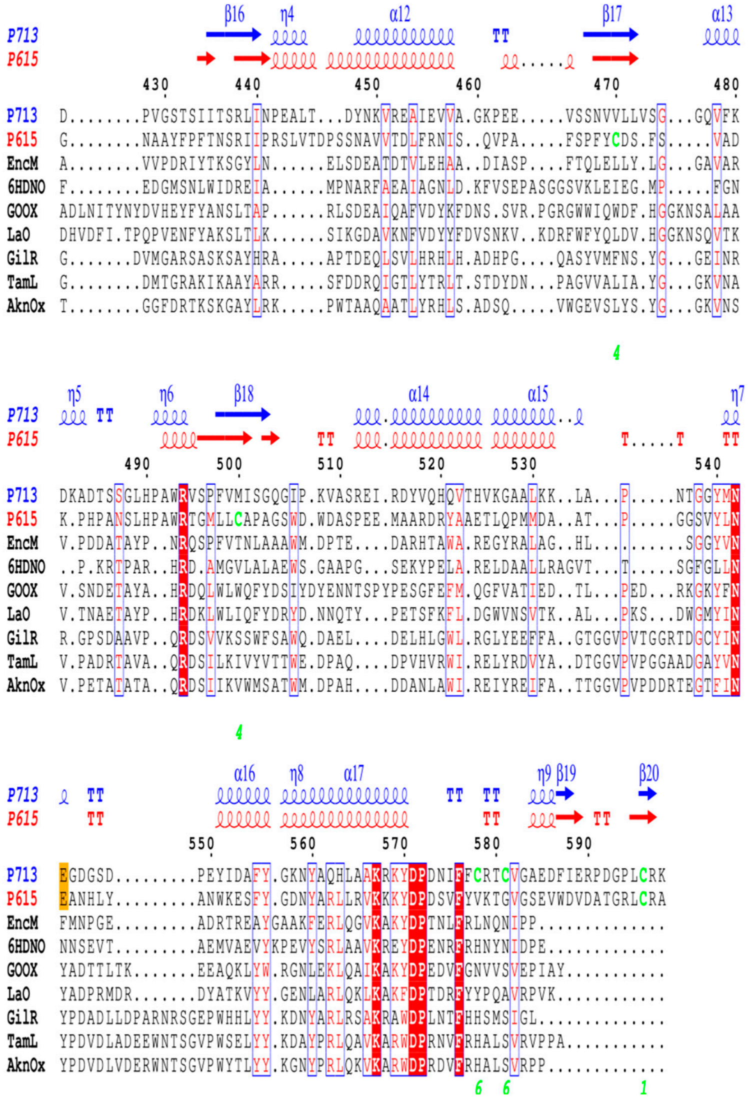

Figure S1: Multiple sequence alignment. Structure–based alignment of MtVAO713 and MtVAO615, EncM (PDB code 3w8w) [22], 6-hydroxy-D-nicotine oxidase (6HDNO, PDB code 2bvf) [23], glucooligosaccharide oxidase (GOOX, PDB code 1ZR6) [4], lactose oxidase (LaO, PDB code 3rj8), oxidoreductase GilR (PDB code 3POP) [25], S. sp. 307-9 tirandamycin oxidase (TamL, PDB code 2Y08) [24] and aclacinomycin oxidoreductase (AckOx, PDB code 2IPI) [8]. The structural alignment was made with Promals3D [38]. The indicated secondary structure elements are those obtained from the crystal structure of MtVAO713 and MtVAO615. Residues involved in FAD binding have a cyan background color, identical residues have a red background color and similar residues have a red color. Residues with orange background are likely to be involved in catalysis. Disulfide linkages are indicated in green italics below the sequences. The figure was created with ESPript [39].

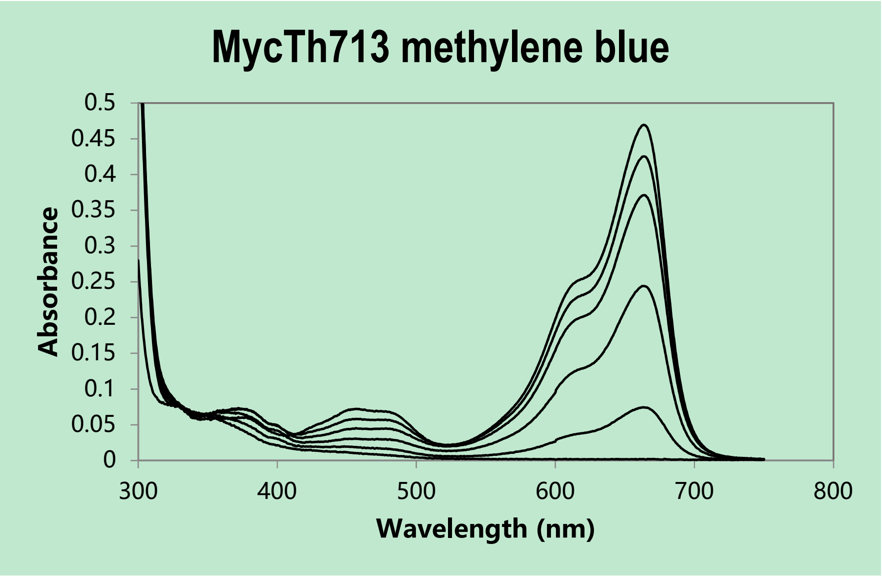

Fig S2: Spectrum of 5 µM MtVAO713 in the redox potential determination experiment with xanthine oxidase. The collected spectra are shown between time point 0 and time point at 34 minutes.

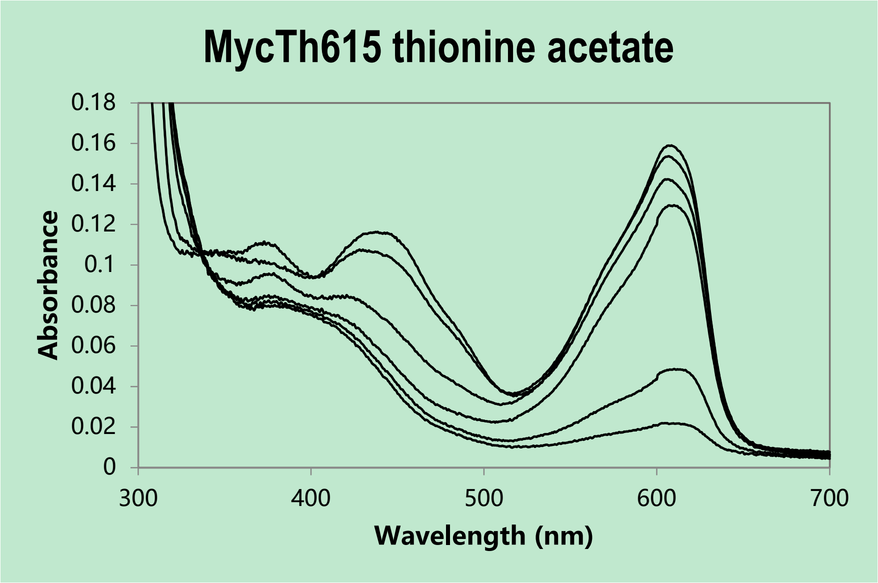

Fig S3: Spectrum of 10 µM MtVAO615 in the redox potential determination experiment with xanthine oxidase. The collected spectra are shown between time point 0 and time point at 88 minutes.

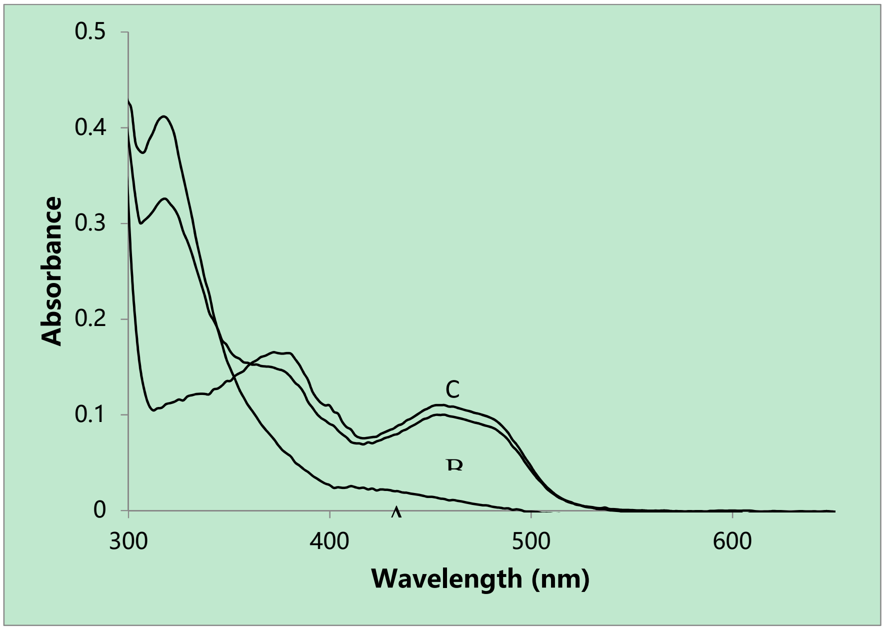

Fig S4: Deconvoluted absorption spectra of reduced (A), intermediate (B) and oxidized (C) MtVAO713 during reoxidation by molecular oxygen as measured by double-mixing stopped-flow spectrophotometry, after a delay time of 1.0 s.

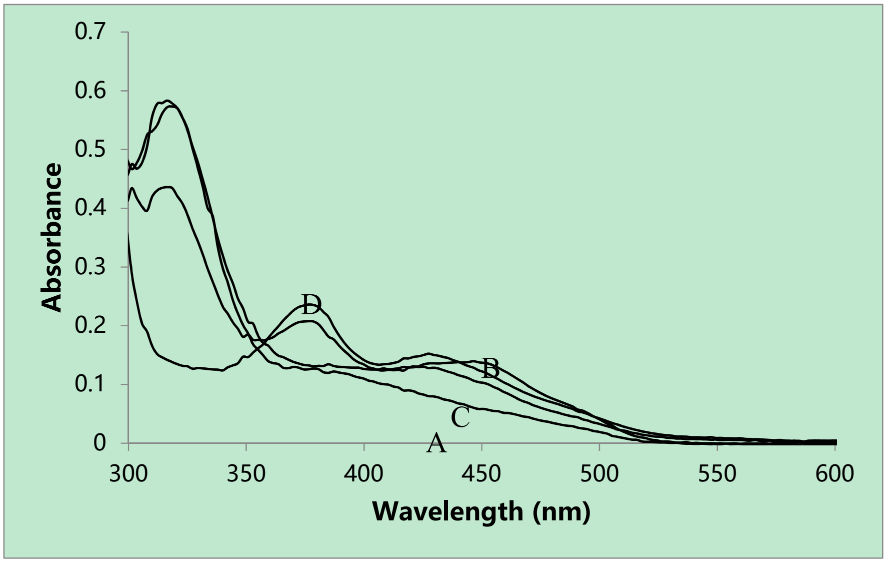

Fig S5: Deconvoluted absorption spectra of reduced (A), intermediate 1 (B), intermediate 2 (C) and oxidized (D) MtVAO615 during reoxidation by molecular oxygen as measured by double-mixing stopped-flow spectrophotometry, after a delay time of 1.0 s.

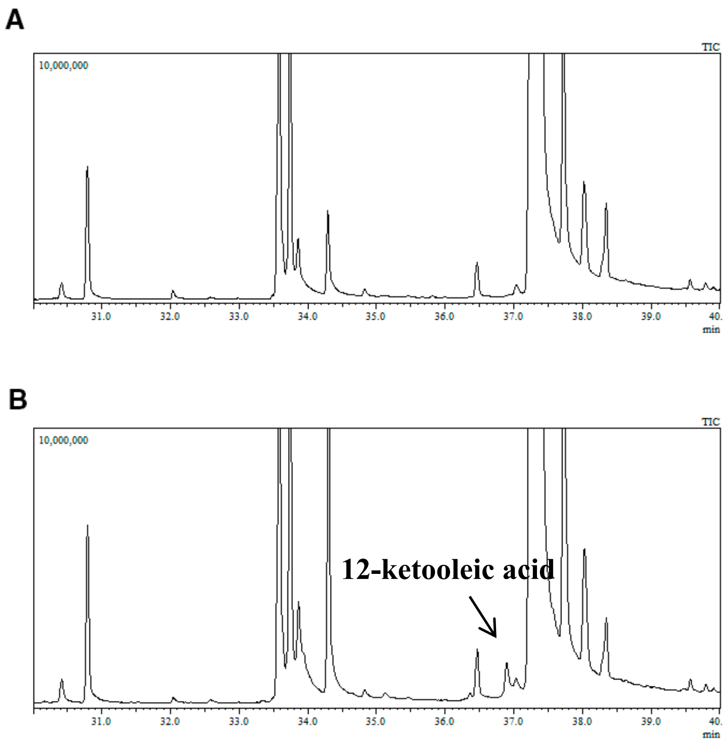

Fig S6: GC spectra of ricinoleic acid conversion by MtVAO713. A) Negative control without MtVAO713. B) Conversion with MtVAO713 present.

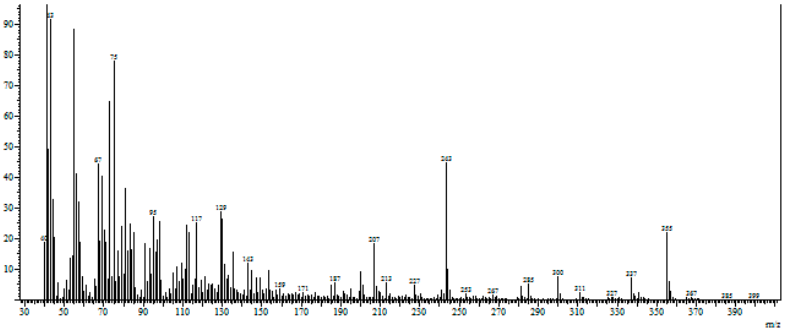

Figure S7: MS spectrum of the selected peak corresponding to 12-ketooleic acid.
